# 3-[(*E*)-(Pyridin-3-yl­imino)­meth­yl]phenol

**DOI:** 10.1107/S1600536812025822

**Published:** 2012-06-13

**Authors:** M. Nawaz Tahir, Akbar Ali, M. Naveed Umar, Ishtiaq Hussain, Hazoor Ahmad Shad

**Affiliations:** a Department of Physics, University of Sargodha, Sargodha, Pakistan; b Department of Chemistry, University of Malakand, Pakistan; c Department of Chemistry, University of Sargodha, Pakistan; d Department of Chemistry, Government Post Graduate College, Gojra, Punjab, Pakistan

## Abstract

Two independent mol­ecules are present in the asymmetric unit of the title compound, C_12_H_10_N_2_O, in which the 3-hy­droxy­benzaldehyde and the pyridin-3-amine units are almost planar [r.m.s. deviations of 0.0236 and 0.0116Å, respectively, in one mol­ecule and 0.0245 and 0.0162Å, respectively, in the other] and are oriented at dihedral angles of 7.21 (7) and 14.77 (7)°. In the crystal, mol­ecules of the same type form inversion dimers *via* pairs of O—H⋯N hydrogen bonds, forming *R*_2_^2^(20) ring motifs. There exist π–π inter­actions between the benzene and pyridine rings of molecules of the same type with centroid–centroid distances of 3.7127 (10) and 3.8439 (10) Å.

## Related literature

For a related structure, see: Wiebcke & Mootz (1982 ▶). For graph-set notation, see: Bernstein *et al.* (1995 ▶).
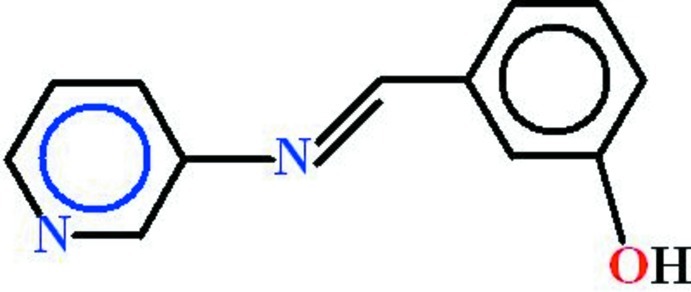


## Experimental

### 

#### Crystal data

C_12_H_10_N_2_O
*M* *_r_* = 198.22
Triclinic, 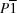

*a* = 5.7768 (5) Å
*b* = 12.1450 (11) Å
*c* = 14.8194 (13) Å
α = 78.207 (4)°
β = 89.641 (3)°
γ = 77.601 (4)°
*V* = 993.26 (15) Å^3^
*Z* = 4
Mo *K*α radiation
μ = 0.09 mm^−1^
*T* = 296 K
0.30 × 0.25 × 0.20 mm

#### Data collection

Bruker Kappa APEXII CCD diffractometer
Absorption correction: multi-scan (*SADABS*; Bruker, 2005 ▶) *T*_min_ = 0.957, *T*_max_ = 0.966
14798 measured reflections
3876 independent reflections
2704 reflections with *I* > 2σ(*I*)
*R*_int_ = 0.029

#### Refinement

*R*[*F* ^2^ > 2σ(*F* ^2^)] = 0.044
*wR*(*F* ^2^) = 0.118
*S* = 1.04
3876 reflections
261 parameters
H-atom parameters constrained
Δρ_max_ = 0.12 e Å^−3^
Δρ_min_ = −0.16 e Å^−3^

### 

Data collection: *APEX2* (Bruker, 2007 ▶); cell refinement: *SAINT* (Bruker, 2007 ▶); data reduction: *SAINT*; program(s) used to solve structure: *SHELXS97* (Sheldrick, 2008 ▶); program(s) used to refine structure: *SHELXL97* (Sheldrick, 2008 ▶); molecular graphics: *ORTEP-3* (Farrugia, 1997 ▶) and *PLATON* (Spek, 2009 ▶); software used to prepare material for publication: *WinGX* (Farrugia, 1999 ▶) and *PLATON*.

## Supplementary Material

Crystal structure: contains datablock(s) global, I. DOI: 10.1107/S1600536812025822/rk2365sup1.cif

Structure factors: contains datablock(s) I. DOI: 10.1107/S1600536812025822/rk2365Isup2.hkl

Supplementary material file. DOI: 10.1107/S1600536812025822/rk2365Isup3.cml

Additional supplementary materials:  crystallographic information; 3D view; checkCIF report

## Figures and Tables

**Table 1:** Hydrogen-bond geometry (Å, °)

*D*—H⋯*A*	*D*—H	H⋯*A*	*D*⋯*A*	*D*—H⋯*A*
O1—H1⋯N2^i^	0.82	2.00	2.810 (2)	172
O2—H2*A*⋯N4^ii^	0.82	1.99	2.8058 (12)	174

Symmetry codes: (i) 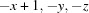; (ii) 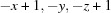.

**Fig. 1.**
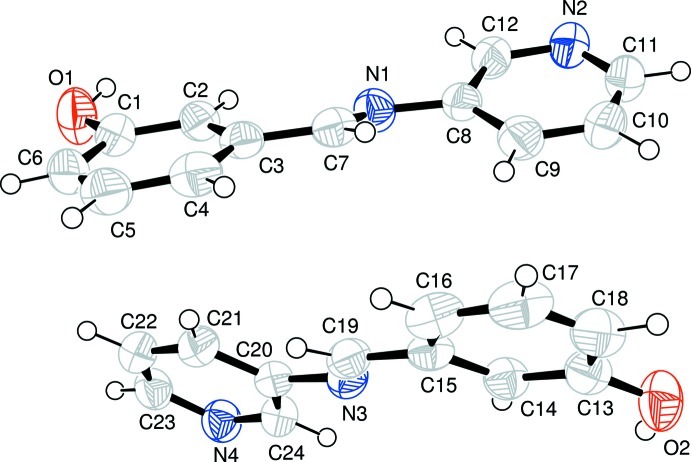
View of the title compound with the atom numbering scheme. The displacement ellipsoids are drawn at the 50% probability level. The H atoms are shown as small circles of arbitrary radii.

**Fig. 2.**
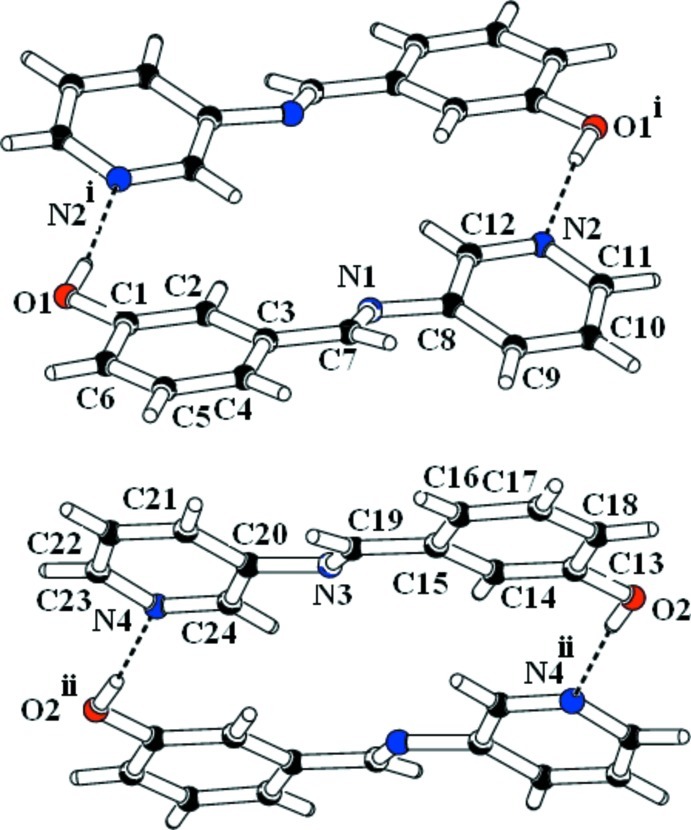
The partial packing which shows that molecules form dimers. Symmetry codes: (i) = -x+1, -y, -z; (ii) = -x+1, -y, -z+1.

## supplementary crystallographic information

### Comment

In the crystal structure, (Fig. 1), of title compound, two molecules in the asymmetric unit are present, which differ slightly from each other geometrically. In one molecule, the 3-hydroxybenzaldehyde group A (C1–C7/O1) and the pyridin-3-amine moiety B (C8–C12/N1/N2) are planar with r.m.s. deviation of 0.0236Å and 0.0116Å, respectively. The dihedral angle between A/B is 14.78 (7)°. In second molecule, the similar groups C (C13–C19/O2) and D (C20–C24/N3/N4) are also planar with r.m.s. deviation of 0.0245Å and 0.0162Å, respectively and the dihedral angle between C/D is 7.21 (7)°. Both molecules are dimerized with themselves due to intermolecular H-bonding of O—H···N type (Table 1, Fig. 2) and form *R*_2_^2^(20) ring motif (Bernstein *et al.*, 1995). There exist π···π interaction between *Cg*1···*Cg*2^iii^ and *Cg*2···*Cg*1^iii^ at a distance of 3.8439 (11)Å. Similarly, there exist π···π interaction between *Cg*3···*Cg*4^iv^ and *Cg*4···*Cg*3^iv^ at a distance of 3.7126 (10)Å. *Cg*1, *Cg*2, *Cg*3 and *Cg*4 are the centroids of (C8–C12/N2), (C1–C6), (C20–C24/N4) and (C13–C18) rings, respectively. Symmetry codes: (iii) = -*x*, -*y*, -*z*; (iv) = -*x*, -*y*, -*z*+1.

The structure of related compounds - *trans*-*N*-benzylidene-3-pyridinamine has been published by Wiebcke & Mootz, 1982.

### Experimental

The title compound has been synthesized as a derivative. Equimolar quantities of 3-hydroxybenzaldehyde and pyridin-3-amine were refluxed in methanol along with few drops of acetic acid as catalyst for 30 min resulting in colourless solution. The solution was kept at room temperature which affoarded colourless prisms after three days.

### Refinement

The H-atoms were positioned geometrically (C—H = 0.93Å, O—H = 0.82Å) and refined as riding with *U*_iso_(H) = *xU*_eq_(C, O), where *x* = 1.5 for hydroxy and *x* = 1.2 for other H-atoms.

### Crystal data

**Table:**

C_12_H_10_N_2_O	*Z* = 4
*M**_r_* = 198.22	*F*(000) = 416
Triclinic, *P*1	*D*_x_ = 1.326 Mg m^−^^3^
Hall symbol: -P 1	Mo *K*α radiation, λ = 0.71073 Å
*a* = 5.7768 (5) Å	Cell parameters from 2704 reflections
*b* = 12.1450 (11) Å	θ = 1.8–26.0°
*c* = 14.8194 (13) Å	µ = 0.09 mm^−^^1^
α = 78.207 (4)°	*T* = 296 K
β = 89.641 (3)°	Prism, colourless
γ = 77.601 (4)°	0.30 × 0.25 × 0.20 mm
*V* = 993.26 (15) Å^3^	

### Data collection

**Table:**

Bruker Kappa APEXII CCD diffractometer	3876 independent reflections
Radiation source: fine-focus sealed tube	2704 reflections with *I* > 2σ(*I*)
Graphite monochromator	*R*_int_ = 0.029
Detector resolution: 8.00 pixels mm^-1^	θ_max_ = 26.0°, θ_min_ = 1.8°
ω–scans	*h* = −6→7
Absorption correction: multi-scan (*SADABS*; Bruker, 2005)	*k* = −14→14
*T*_min_ = 0.957, *T*_max_ = 0.966	*l* = −18→18
14798 measured reflections	

### Refinement

**Table:**

Refinement on *F*^2^	Primary atom site location: structure-invariant direct methods
Least-squares matrix: full	Secondary atom site location: difference Fourier map
*R*[*F*^2^ > 2σ(*F*^2^)] = 0.044	Hydrogen site location: inferred from neighbouring sites
*wR*(*F*^2^) = 0.118	H-atom parameters constrained
*S* = 1.04	*w* = 1/[σ^2^(*F*_o_^2^) + (0.0494*P*)^2^ + 0.1521*P*] where *P* = (*F*_o_^2^ + 2*F*_c_^2^)/3
3876 reflections	(Δ/σ)_max_ < 0.001
261 parameters	Δρ_max_ = 0.12 e Å^−^^3^
0 restraints	Δρ_min_ = −0.16 e Å^−^^3^

### Special details

**Table:**

Geometry. All s.u.'s (except the s.u. in the dihedral angle between two l.s. planes) are estimated using the full covariance matrix. The cell s.u.'s are taken into account individually in the estimation of s.u.'s in distances, angles and torsion angles; correlations between s.u.'s in cell parameters are only used when they are defined by crystal symmetry. An approximate (isotropic) treatment of cell s.u.'s is used for estimating s.u.'s involving l.s. planes.
Refinement. Refinement of *F*^2^ against ALL reflections. The weighted *R*-factor *wR* and goodness of fit *S* are based on *F*^2^, conventional *R*-factors *R* are based on *F*, with *F* set to zero for negative *F*^2^. The threshold expression of *F*^2^ > σ(*F*^2^) is used only for calculating *R*-factors(gt) *etc*. and is not relevant to the choice of reflections for refinement. *R*-factors based on *F*^2^ are statistically about twice as large as those based on *F*, and *R*-factors based on ALL data will be even larger.

### Fractional atomic coordinates and isotropic or equivalent isotropic displacement parameters (Å^2^)

**Table:**

	*x*	*y*	*z*	*U*_iso_*/*U*_eq_	
O1	0.3002 (3)	−0.38329 (11)	0.13058 (10)	0.0720 (6)	
N1	0.1603 (3)	0.05071 (12)	0.10067 (9)	0.0500 (5)	
N2	0.4028 (3)	0.29991 (13)	0.00539 (10)	0.0566 (6)	
C1	0.1241 (3)	−0.29847 (16)	0.15019 (12)	0.0525 (6)	
C2	0.1340 (3)	−0.18383 (14)	0.12760 (11)	0.0470 (6)	
C3	−0.0509 (3)	−0.09881 (15)	0.14672 (11)	0.0453 (6)	
C4	−0.2498 (3)	−0.13042 (17)	0.18808 (12)	0.0562 (7)	
C5	−0.2555 (3)	−0.24564 (19)	0.21309 (13)	0.0622 (8)	
C6	−0.0716 (3)	−0.32902 (17)	0.19536 (12)	0.0603 (7)	
C7	−0.0320 (3)	0.02167 (15)	0.12514 (11)	0.0478 (6)	
C8	0.1768 (3)	0.16709 (14)	0.08358 (11)	0.0450 (5)	
C9	0.0388 (3)	0.25436 (16)	0.12042 (12)	0.0574 (7)	
C10	0.0867 (4)	0.36199 (16)	0.09900 (13)	0.0642 (7)	
C11	0.2672 (3)	0.38158 (16)	0.04216 (13)	0.0609 (7)	
C12	0.3563 (3)	0.19524 (15)	0.02750 (12)	0.0506 (6)	
O2	−0.01543 (13)	0.39654 (6)	0.38845 (7)	0.0683 (5)	
N3	0.19245 (12)	−0.03508 (6)	0.39821 (6)	0.0500 (5)	
N4	0.64794 (12)	−0.29360 (6)	0.47831 (6)	0.0537 (5)	
C13	−0.12229 (13)	0.31831 (6)	0.36181 (7)	0.0512 (6)	
C14	−0.02002 (12)	0.20228 (6)	0.37886 (6)	0.0467 (6)	
C15	−0.1372 (3)	0.12369 (15)	0.35433 (11)	0.0451 (6)	
C16	−0.3621 (3)	0.16340 (18)	0.31206 (12)	0.0573 (7)	
C17	−0.4595 (3)	0.28010 (19)	0.29175 (13)	0.0638 (7)	
C18	−0.3421 (3)	0.35724 (17)	0.31563 (12)	0.0598 (7)	
C19	−0.0232 (3)	0.00155 (15)	0.37155 (11)	0.0480 (6)	
C20	0.3013 (3)	−0.15308 (14)	0.41181 (11)	0.0436 (5)	
C21	0.2182 (3)	−0.23740 (15)	0.37925 (12)	0.0516 (6)	
C22	0.3527 (3)	−0.34747 (15)	0.39586 (12)	0.0553 (7)	
C23	0.5661 (3)	−0.37225 (15)	0.44406 (12)	0.0547 (6)	
C24	0.5160 (3)	−0.18691 (15)	0.46091 (12)	0.0491 (6)	
H1	0.38859	−0.35466	0.09392	0.1080*	
H2	0.26668	−0.16302	0.09910	0.0564*	
H4	−0.37822	−0.07438	0.19884	0.0674*	
H5	−0.38665	−0.26689	0.24252	0.0747*	
H6	−0.07728	−0.40633	0.21353	0.0723*	
H7	−0.16522	0.07872	0.12950	0.0574*	
H9	−0.08377	0.23995	0.15891	0.0689*	
H10	−0.00331	0.42169	0.12306	0.0771*	
H11	0.29688	0.45537	0.02849	0.0732*	
H12	0.45151	0.13687	0.00348	0.0606*	
H2A	0.09555	0.36277	0.42494	0.1024*	
H14	0.13035	0.17617	0.40734	0.0560*	
H16	−0.44634	0.11167	0.29759	0.0688*	
H17	−0.60742	0.30686	0.26131	0.0765*	
H18	−0.40970	0.43573	0.30089	0.0717*	
H19	−0.11186	−0.05113	0.36265	0.0576*	
H21	0.07331	−0.21935	0.34665	0.0620*	
H22	0.29991	−0.40511	0.37463	0.0663*	
H23	0.65766	−0.44690	0.45330	0.0656*	
H24	0.57225	−0.13110	0.48342	0.0589*	

### Atomic displacement parameters (Å^2^)

**Table:**

	*U*^11^	*U*^22^	*U*^33^	*U*^12^	*U*^13^	*U*^23^
O1	0.0813 (10)	0.0476 (8)	0.0915 (11)	−0.0207 (7)	0.0199 (8)	−0.0179 (7)
N1	0.0536 (9)	0.0454 (9)	0.0502 (9)	−0.0099 (7)	0.0086 (7)	−0.0094 (7)
N2	0.0648 (9)	0.0486 (10)	0.0605 (10)	−0.0178 (8)	0.0029 (7)	−0.0147 (8)
C1	0.0600 (11)	0.0510 (11)	0.0503 (11)	−0.0189 (9)	0.0007 (8)	−0.0123 (9)
C2	0.0508 (10)	0.0507 (11)	0.0430 (10)	−0.0191 (8)	0.0057 (7)	−0.0093 (8)
C3	0.0466 (9)	0.0554 (11)	0.0358 (9)	−0.0146 (8)	−0.0006 (7)	−0.0099 (8)
C4	0.0474 (10)	0.0757 (14)	0.0498 (11)	−0.0184 (9)	0.0040 (8)	−0.0180 (10)
C5	0.0592 (12)	0.0834 (16)	0.0531 (12)	−0.0352 (11)	0.0082 (9)	−0.0145 (10)
C6	0.0728 (13)	0.0638 (13)	0.0529 (12)	−0.0372 (11)	0.0017 (9)	−0.0083 (9)
C7	0.0481 (10)	0.0534 (11)	0.0406 (10)	−0.0049 (8)	0.0003 (8)	−0.0132 (8)
C8	0.0507 (9)	0.0424 (10)	0.0400 (9)	−0.0059 (8)	−0.0024 (7)	−0.0087 (8)
C9	0.0662 (12)	0.0530 (12)	0.0500 (11)	−0.0053 (9)	0.0103 (9)	−0.0120 (9)
C10	0.0836 (14)	0.0471 (12)	0.0605 (12)	−0.0029 (10)	0.0044 (10)	−0.0198 (10)
C11	0.0778 (13)	0.0447 (11)	0.0613 (12)	−0.0143 (10)	−0.0061 (10)	−0.0120 (9)
C12	0.0550 (10)	0.0472 (11)	0.0514 (11)	−0.0103 (8)	0.0046 (8)	−0.0156 (8)
O2	0.0658 (8)	0.0451 (8)	0.0890 (10)	−0.0024 (6)	−0.0092 (7)	−0.0127 (7)
N3	0.0533 (9)	0.0449 (9)	0.0511 (9)	−0.0079 (7)	−0.0042 (7)	−0.0112 (7)
N4	0.0559 (9)	0.0460 (9)	0.0565 (9)	−0.0070 (7)	0.0029 (7)	−0.0089 (7)
C13	0.0488 (10)	0.0517 (11)	0.0492 (10)	−0.0036 (8)	0.0047 (8)	−0.0096 (8)
C14	0.0430 (9)	0.0482 (11)	0.0441 (10)	−0.0014 (8)	−0.0026 (7)	−0.0075 (8)
C15	0.0454 (9)	0.0539 (11)	0.0352 (9)	−0.0067 (8)	0.0054 (7)	−0.0121 (8)
C16	0.0438 (10)	0.0779 (14)	0.0521 (11)	−0.0082 (9)	0.0017 (8)	−0.0230 (10)
C17	0.0444 (10)	0.0849 (16)	0.0539 (12)	0.0083 (10)	−0.0056 (8)	−0.0191 (11)
C18	0.0546 (11)	0.0592 (12)	0.0540 (11)	0.0103 (9)	0.0030 (9)	−0.0092 (10)
C19	0.0526 (10)	0.0538 (11)	0.0422 (10)	−0.0158 (8)	0.0053 (8)	−0.0159 (8)
C20	0.0506 (9)	0.0428 (10)	0.0385 (9)	−0.0111 (8)	0.0044 (7)	−0.0100 (8)
C21	0.0586 (10)	0.0509 (11)	0.0470 (10)	−0.0135 (9)	−0.0027 (8)	−0.0122 (8)
C22	0.0721 (12)	0.0460 (11)	0.0512 (11)	−0.0168 (9)	0.0018 (9)	−0.0140 (9)
C23	0.0675 (12)	0.0404 (10)	0.0540 (11)	−0.0093 (9)	0.0101 (9)	−0.0075 (8)
C24	0.0526 (10)	0.0442 (10)	0.0521 (11)	−0.0122 (8)	0.0020 (8)	−0.0122 (8)

### Geometric parameters (Å, º)

**Table:**

O1—C1	1.360 (2)	C6—H6	0.9300
O1—H1	0.8200	C7—H7	0.9300
O2—C13	1.3594 (11)	C9—H9	0.9300
O2—H2A	0.8200	C10—H10	0.9300
N1—C7	1.265 (2)	C11—H11	0.9300
N1—C8	1.408 (2)	C12—H12	0.9300
N2—C11	1.333 (2)	C13—C14	1.3775 (11)
N2—C12	1.331 (2)	C13—C18	1.388 (2)
N3—C20	1.4094 (19)	C14—C15	1.3870 (19)
N3—C19	1.2658 (19)	C15—C16	1.389 (3)
N4—C23	1.3374 (19)	C15—C19	1.459 (3)
N4—C24	1.330 (2)	C16—C17	1.379 (3)
C1—C6	1.390 (3)	C17—C18	1.369 (3)
C1—C2	1.377 (3)	C20—C21	1.389 (2)
C2—C3	1.389 (2)	C20—C24	1.381 (2)
C3—C4	1.388 (3)	C21—C22	1.369 (3)
C3—C7	1.460 (3)	C22—C23	1.373 (3)
C4—C5	1.380 (3)	C14—H14	0.9300
C5—C6	1.366 (3)	C16—H16	0.9300
C8—C12	1.381 (2)	C17—H17	0.9300
C8—C9	1.388 (3)	C18—H18	0.9300
C9—C10	1.368 (3)	C19—H19	0.9300
C10—C11	1.366 (3)	C21—H21	0.9300
C2—H2	0.9300	C22—H22	0.9300
C4—H4	0.9300	C23—H23	0.9300
C5—H5	0.9300	C24—H24	0.9300
			
C1—O1—H1	109.00	C10—C11—H11	118.00
C13—O2—H2A	109.00	C8—C12—H12	118.00
C7—N1—C8	120.93 (16)	N2—C12—H12	118.00
C11—N2—C12	116.56 (16)	O2—C13—C18	118.56 (11)
C19—N3—C20	121.28 (12)	C14—C13—C18	119.06 (11)
C23—N4—C24	116.83 (12)	O2—C13—C14	122.38 (8)
C2—C1—C6	119.03 (17)	C13—C14—C15	121.21 (10)
O1—C1—C6	118.63 (17)	C14—C15—C19	120.14 (14)
O1—C1—C2	122.33 (16)	C16—C15—C19	120.83 (17)
C1—C2—C3	121.07 (16)	C14—C15—C16	119.02 (16)
C2—C3—C7	119.93 (16)	C15—C16—C17	119.49 (18)
C2—C3—C4	119.13 (17)	C16—C17—C18	121.11 (17)
C4—C3—C7	120.93 (17)	C13—C18—C17	119.99 (17)
C3—C4—C5	119.48 (18)	N3—C19—C15	122.14 (15)
C4—C5—C6	121.09 (17)	N3—C20—C24	115.98 (14)
C1—C6—C5	120.09 (19)	C21—C20—C24	117.11 (16)
N1—C7—C3	121.91 (16)	N3—C20—C21	126.89 (15)
C9—C8—C12	117.15 (16)	C20—C21—C22	119.00 (16)
N1—C8—C12	116.06 (15)	C21—C22—C23	119.57 (17)
N1—C8—C9	126.72 (16)	N4—C23—C22	122.80 (16)
C8—C9—C10	118.67 (17)	N4—C24—C20	124.66 (16)
C9—C10—C11	119.83 (18)	C13—C14—H14	119.00
N2—C11—C10	123.09 (18)	C15—C14—H14	119.00
N2—C12—C8	124.68 (17)	C15—C16—H16	120.00
C3—C2—H2	119.00	C17—C16—H16	120.00
C1—C2—H2	119.00	C16—C17—H17	119.00
C3—C4—H4	120.00	C18—C17—H17	119.00
C5—C4—H4	120.00	C13—C18—H18	120.00
C4—C5—H5	119.00	C17—C18—H18	120.00
C6—C5—H5	119.00	N3—C19—H19	119.00
C1—C6—H6	120.00	C15—C19—H19	119.00
C5—C6—H6	120.00	C20—C21—H21	120.00
N1—C7—H7	119.00	C22—C21—H21	121.00
C3—C7—H7	119.00	C21—C22—H22	120.00
C8—C9—H9	121.00	C23—C22—H22	120.00
C10—C9—H9	121.00	N4—C23—H23	119.00
C11—C10—H10	120.00	C22—C23—H23	119.00
C9—C10—H10	120.00	N4—C24—H24	118.00
N2—C11—H11	118.00	C20—C24—H24	118.00
			
C8—N1—C7—C3	−177.57 (15)	C12—C8—C9—C10	0.4 (3)
C7—N1—C8—C9	25.4 (3)	C9—C8—C12—N2	−1.2 (3)
C7—N1—C8—C12	−157.68 (16)	N1—C8—C12—N2	−178.40 (16)
C12—N2—C11—C10	−0.7 (3)	C8—C9—C10—C11	0.2 (3)
C11—N2—C12—C8	1.3 (3)	C9—C10—C11—N2	0.0 (3)
C20—N3—C19—C15	−178.07 (14)	O2—C13—C14—C15	177.30 (11)
C19—N3—C20—C21	16.4 (2)	C18—C13—C14—C15	−2.84 (17)
C19—N3—C20—C24	−165.28 (15)	O2—C13—C18—C17	−176.90 (14)
C23—N4—C24—C20	1.1 (2)	C14—C13—C18—C17	3.2 (2)
C24—N4—C23—C22	−2.3 (2)	C13—C14—C15—C16	−0.2 (2)
C6—C1—C2—C3	−2.0 (3)	C13—C14—C15—C19	178.83 (12)
O1—C1—C6—C5	−177.63 (17)	C14—C15—C16—C17	2.8 (2)
O1—C1—C2—C3	178.60 (16)	C19—C15—C16—C17	−176.18 (16)
C2—C1—C6—C5	2.9 (3)	C14—C15—C19—N3	−8.8 (2)
C1—C2—C3—C4	−0.9 (3)	C16—C15—C19—N3	170.20 (15)
C1—C2—C3—C7	177.73 (16)	C15—C16—C17—C18	−2.5 (3)
C2—C3—C7—N1	−9.6 (2)	C16—C17—C18—C13	−0.6 (3)
C4—C3—C7—N1	169.05 (16)	N3—C20—C21—C22	177.27 (15)
C2—C3—C4—C5	2.9 (3)	C24—C20—C21—C22	−1.0 (2)
C7—C3—C4—C5	−175.76 (16)	N3—C20—C24—N4	−177.97 (14)
C3—C4—C5—C6	−2.0 (3)	C21—C20—C24—N4	0.5 (3)
C4—C5—C6—C1	−1.0 (3)	C20—C21—C22—C23	−0.1 (3)
N1—C8—C9—C10	177.26 (17)	C21—C22—C23—N4	1.8 (3)

### Hydrogen-bond geometry (Å, º)

**Table:**

*D*—H···*A*	*D*—H	H···*A*	*D*···*A*	*D*—H···*A*
O1—H1···N2^i^	0.82	2.00	2.810 (2)	172
O2—H2*A*···N4^ii^	0.82	1.99	2.8058 (12)	174

Symmetry codes: (i) −*x*+1, −*y*, −*z*; (ii) −*x*+1, −*y*, −*z*+1.
